# The Efficacy of Mitapivat in Case of Transfusion Dependence Linked to Beta Thalassemia Trait Associated with Pyruvate Kinase Deficiency

**DOI:** 10.1111/jcmm.70217

**Published:** 2024-11-25

**Authors:** Carmina Fatigati, Silvia Costantini, Anna Spasiano, Brunella Ziello, Antonella Gambale, Paolo Ricchi

**Affiliations:** ^1^ Rare Red Blood Cells Diseases Unit AORN A. Cardarelli Naples Italy; ^2^ U.O.C. Genetica Medica A.O.U. Federico II Naples Italy; ^3^ Department of Molecular Medicine and Medical Biotechnology University Federico II Naples Italy

**Keywords:** hemoglobinopathies, hemolytic anaemia, new agents, transfusion‐ red blood cells

To the Editor

Recent reports have highlighted cases where heterozygosity for a haemoglobin subunit beta (HBB) variant alone does not fully account for the clinical phenotype. These cases are characterised by varying degrees of anaemia (sometimes requiring transfusion), splenomegaly, abnormal hemolytic indices, and liver hemosiderosis. Many have been linked to the co‐presence of red blood cell membrane disorders [1]. Here, we present a unique case of beta‐thalassemia trait combined with Pyruvate Kinase (PK) deficiency, detailing the diagnostic challenges and the patient's successful treatment. The patient gave full permission for the publication of his history and his clinical data in anonymous form.

The patient first presented to our unit in 2013, at the age of 40, for a consultation regarding his chronic non‐immune hemolytic anaemia. He had been evaluated for anaemia since early childhood and was diagnosed with heterozygous beta‐thalassemia due to the β39 C → T mutation at the age of two. From ages 2–7, he received multiple red blood cell transfusions. Recurrent hemolytic episodes occurred, particularly after infections or stress. His medical history also includes portal cavernoma with oesophageal varices and severe obstructive sleep apnoea syndrome. At age 37, he underwent laparoscopic cholecystectomy and splenectomy following cholecystitis. His haemoglobin levels ranged from 7.5 g/dL before the splenectomy to 8.3 g/dL afterward.

Table 1 summarises the key biochemical parameters at our initial evaluation. We confirmed the presence of the unique HBB mutation (β39 C → T) and after excluding the co‐existence of alpha globin genes defects (deletion, variant and multiplication) and red blood cell membrane disorders, PK deficiency was suspected based on earlier biochemical tests showing low PK activity. His medical history and clinical and biochemical signs, including reduced bone density, hepatomegaly, jaundice, increased reticulocytes, indirect hyperbilirubinemia, and mild hyperferritinemia, also supported this suspicion. In 2015, molecular testing confirmed PK deficiency caused by a homozygous missense mutation (c.1456 C → T) in the LR‐PK gene.

**TABLE 1:** Haematological parameters.

	Baseline	During transfusion regimen	At 6 months of mitapivat treatment	At 12 months of mitapivat treatment
Haemoglobin gr/dL	8.3	9.6 (9.8–9.4)^a^	10.2	9.7
Reticulocytes 10^6^/μL	0.56	0.56	0.43	0.42
LDH UI/L	441	304 (482–193)	286	332
Urate mg/dL	4.7	3.6 (4.9–1.6)	3.2	3.7
Indirect bilirubin mg/dL	4.6	3.3 (6.1–1.69)	2.08	2.19
sTfR mg/L	8.74	6.27 (9.54–4.41)	6.7	5.99
MCV fL	69.5	79.3 (86.8–68.8)	70.1	74.1
NRBC 10^3^/μL	3.2	1.6 (2.6–0.9)	2.1	1.7
Mg Fe Kg/die^b^		0.17 (0.26–0.07)		

*Note:* Table 1 shows main haematological parameters observed during each step of patient management. Data are expressed as mean and min‐max values are reported in brackets.

Abbreviations: LDH, lactic dehydrogenase; MCV, mean cell volume; NRBC, nucleated red blood cells; sTfR, soluble transferrin receptor.

^a^ Pre‐transfusion value.

^b^ Mean annual iron intake.

Due to worsening dyspnoea and echocardiographic findings of increased pulmonary artery pressure and atrial volume, regular red blood cell transfusions (BT) were initiated in 2014. Since then, the patient has received consistent transfusions, with mean pre‐transfusion haemoglobin levels of approximately 9.6 g/dL and post‐transfusion levels of around 12 g/dL (data not shown). Table 1 outlines the haematological parameters before and after the transfusion regimen.

Following the success of the ACTIVATE‐T trial [2], mitapivat, a targeted PK activator, became available in Italy through an Expanded Access Program provided by the manufacturer (Agios Pharmaceuticals). In July 2023 and on the occasion of the last transfusion administered, after receiving approval from the local ethics committee, the patient began treatment with Mitapivat at a dose of 5 mg twice daily. After 4 weeks, the dosage was increased to 20 mg twice daily and thereafter to 50 mg twice daily, as recommended. Following treatment with mitapivat, the patient achieved an early and sustained independence from red blood cell transfusions. Despite the absence of BT, during 1 year of follow up, his average haemoglobin level was 10.2 g/dL and 9.7 g/day, at 6 and 12 months of treatment, respectively, being the increase in Hb more than 1 g/L as compared to baseline. Table 1 also shows the effects of mitapivat on key biochemical parameters. Notably, the improvements in markers of haemolysis and erythropoietic activity were more pronounced during mitapivat treatment compared to those observed during BT. Overall, the treatment was well tolerated, with no adverse events reported; the patient experienced only mild insomnia, and there was no significant increase in transaminase levels with respect to baseline levels (data not shown).

The diagnosis, management, and treatment of patients with chronic non‐immune hemolytic anaemia who are only carriers of the beta‐thalassemia trait and have been managed for years only with occasional red cell blood transfusions is an evolving field of research. This case represents the first documented instance of PK deficiency in conjunction with beta‐thalassemia trait, where proper identification was crucial for the successful treatment approach given the advent of a new oral therapeutic option for patients with PK deficiency, bearing in mind that mitapivat is not available or approved for thalassemia and has not been studied in subject with thalassemia trait. Red cell PK deficiency is the most common cause of chronic hereditary nonspherocytic hemolytic anaemia and the most frequent enzyme abnormality of the glycolytic pathway [3]. Mitapivat, an oral PK activator recently approved for the treatment of PK deficiency, has been shown to significantly improve its clinical manifestations and enhance quality of life [2, 4]. Moreover, according to the latest results of ENERGIZE study, mitapivat also demonstrated effectiveness in counteracting ineffective erythropoiesis and increasing red blood cell survival in patients with thalassemia, revealing a statistically significant improvement in haemoglobin (Hb) response compared to placebo [5]. It is unclear to what extent the presence of beta‐thalassemia trait may have contributed to the clinical presentation in this patient, but data from this case report suggest that, in case of PK deficiency, the presence of thalassemia mutation likely does not limit the ability of mitapivat to correct and sustain PK activity. This resulted in improvement in haemoglobin, haemolysis and erythropoiesis without any safety concerns. Additional studies are needed to better understand the impact of mitapivat in the setting of non‐immune haemolytic anaemia stemming from multiple mutation in diseases related genes.

## Author Contributions

**Carmina Fatigati:** data curation (lead), writing – review and editing (equal). **Silvia Costantini:** data curation (equal), writing – review and editing (equal). **Anna Spasiano:** data curation (equal), writing – review and editing (equal). **Brunella Ziello:** resources (equal), software (equal). **Antonella Gambale:** methodology (equal), project administration (equal). **Paolo Ricchi:** conceptualization (lead), writing – review and editing (lead).

## Consent

The patient gave full permission for the publication of his history and his clinical data in anonymous form.

## Conflicts of Interest

Paolo Ricchi received honoraria/payment for lectures and presentations, from Agios Pharmaceuticals and Bristol Myers Squibb; Advisory Board for Agios Pharmaceuticals and BMS. All other authors declare that they do not have any conflict of interest.

## Data Availability

The data that support the findings of this study are available from the corresponding author upon reasonable request.
